# Neurological Comorbidity Burden, Outcomes, and Disparities in Head and Neck Cancer During COVID-19

**DOI:** 10.3390/reports9030218

**Published:** 2026-07-09

**Authors:** Narayan Dhimal, Roberto Pili, Joel B. Epstein, Vipanchika Satheeshkumar, Minu Ponnamma Mohan, Kapil Meleveedu, Poolakkad S. Satheeshkumar

**Affiliations:** 1 Jacobs School of Medicine and Biomedical Sciences, University at Buffalo, Buffalo, NY 14203, USA; 2 Division of Hematology and Oncology, Department of Medicine, Jacobs School of Medicine and Biomedical Sciences, University at Buffalo, Buffalo, NY 14203, USA; 3 City of Hope Comprehensive Cancer Center, Duarte, CA 91010, USA; 4 Cedars-Sinai Medical Center, Los Angeles, CA 90048, USA; 5 Department of Psychiatry, Jacobs School of Medicine and Biomedical Sciences, University at Buffalo, Buffalo, NY 14203, USA; 6 Carol and Ray Neag Comprehensive Cancer Center, University of Connecticut Health Center, Farmington, CT 06030, USA

**Keywords:** head and neck cancer, neurological complications, hospitalization, health disparities, National Inpatient Sample, mortality

## Abstract

**Background:** Neurological complications (NCs) are increasingly recognized as contributors to adverse outcomes in hospitalized cancer populations, yet their burden and associated disparities in patients with head and neck cancer (HNC) remain poorly characterized. This study evaluated the association between NCs and hospital outcomes in HNC and examined sociodemographic disparities. **Methods:** A retrospective cross-sectional study was conducted using the 2021 National Inpatient Sample, a nationally representative database of U.S. hospitalizations. Adult patients with a primary diagnosis of HNC were included. NCs were identified using ICD-10-CM codes. Survey-weighted multivariable regression models assessed associations with outcomes. **Results:** Among 57,615 weighted HNC hospitalizations, corresponding to 11,523 unweighted discharges, 6320 (unweighted *n* = 1328; 11%) had at least one NC. NCs were independently associated with higher hospital charges (adjusted geometric mean ratio [aGMR], 1.38, 95% CI 1.26–1.51), longer length of stay (aGMR, 1.25, 95% CI 1.17–1.34), and increased in-hospital mortality (aOR 2.42, 95% CI 1.96–2.98). NCs were also associated with higher odds of hospital-acquired complications (aOR 1.92), septicemia (1.90), fluid and electrolyte disorders (1.65), COVID-19 infection (1.66), and emergency department admission (1.33). Disparities were observed, with Hispanic and Other race patients incurring higher charges and Black and Hispanic patients experiencing longer hospital stays; Medicaid and self-pay patients had higher mortality compared with those on Medicare. **Conclusions:** NCs are associated with worse outcomes and increased healthcare utilization among hospitalized HNC patients; however, given NIS limitations, including lack of cancer stage, treatment history, performance status, and brain metastasis data, these findings should be interpreted as non-causal associations. Early NC recognition and disparity-focused interventions may improve inpatient cancer care.

## 1. Introduction

Head and neck cancer (HNC) represents a significant global public health burden, accounting for over 65,000 new cases annually in the United States and contributing to substantial morbidity, healthcare costs, and mortality [1,2,3]. Advances in multimodal therapy have improved survival for many patients with HNC, resulting in a growing population of cancer survivors who experience long-term functional and neurocognitive sequelae related to both disease and treatment [1,4]. The complexity of treatment, including surgery, radiation, and chemotherapy, places patients at elevated risk for acute complications and prolonged hospitalizations [1,2]. During hospitalization, HNC patients may be especially susceptible to acute neurocognitive syndromes because of airway compromise, high symptom burden requiring sedatives or opioids, malnutrition and dehydration, infection risk, and treatment-related neurotoxicity from radiotherapy with or without systemic chemotherapy [1]. Neurologic complications and delirium have been well described across broader oncology populations, including patients with hematologic malignancies and those undergoing surgery for solid tumors, where they are consistently associated with longer hospitalizations, higher costs, and increased mortality [5,6,7]. However, although such complications have been examined in selected surgical oncology cohorts [5,6,7,8,9,10], few studies have quantified their burden among hospitalized patients with HNC using nationally representative data, where early recognition and management may improve outcomes [9]. Furthermore, limited evidence exists regarding how sociodemographic factors such as race, insurance status, and geographic location influence outcomes in this high-risk population [11,12].

The COVID-19 pandemic has further disrupted cancer care delivery and exacerbated risks for hospitalized patients [13,14,15,16]. Immunosuppression, treatment delays, and infection-related complications have disproportionately affected oncology populations, including those with HNC [13,17,18]. Moreover, the pandemic has amplified existing disparities in access to care, with racial and ethnic minorities, uninsured patients, and those from rural or low-income communities facing greater barriers to timely diagnosis and treatment [11,12]. In this setting, hospitalized patients with HNC may face heightened vulnerability to neurological complications and related adverse outcomes, underscoring the importance of examining these associations during the COVID-19 era.

Therefore, we conducted a retrospective analysis of the 2021 National Inpatient Sample to evaluate the association of neurological complications, adverse in-hospital outcomes, and healthcare utilization among patients hospitalized with head and neck cancer [19]. We examined disparities across race/ethnicity, insurance type, income level, and rurality to identify vulnerable subgroups to inform focused interventions.

Understanding the impact of neurological complications on inpatient outcomes in HNC is essential for developing targeted strategies to reduce morbidity, improve quality of care, and address health outcomes.

## 2. Methods

### 2.1. Study Design and Data Source

We conducted a retrospective cross-sectional analysis using the 2021 National Inpatient Sample (NIS), the largest publicly available all-payer inpatient healthcare database in the United States [19]. The NIS is maintained by the Healthcare Cost and Utilization Project (HCUP) [20] and includes a 20% stratified sample of discharges from U.S. community hospitals, weighted to produce national estimates. Each record contains patient demographics, hospital characteristics, diagnoses, procedures, and outcomes. Diagnoses and procedures were identified using the International Classification of Diseases, Tenth Revision, Clinical Modification and Procedure Coding System (ICD-10-CM/PCS) [21]. This study was conducted and reported in accordance with the EQUATOR Network recommendations using the STROBE reporting guideline for cross-sectional studies. This study was deemed exempt from institutional review board oversight due to the use of de-identified publicly available data.

### 2.2. Study Population

We identified adult hospitalizations (age ≥ 18 years) with a primary diagnosis of head and neck cancer (HNC) using ICD-10-CM codes corresponding to malignant neoplasms of the oral cavity, pharynx, larynx, and related structures. Neurological comorbidity burden was identified using ICD-10-CM codes defined within the Elixhauser Comorbidity Software Refined for ICD-10-CM (v2026.1, Rockville, MD, USA), specifically the category of “other neurological disorders,” used as a proxy to capture a broad range of pre-existing neurological conditions relevant to clinical outcomes [19,20]. Because the NIS is discharge-level administrative data, these codes do not allow determination of neurological symptom severity, timing of onset, or whether the condition was present on admission versus developed during hospitalization. Hospitalizations with missing key demographic or outcome variables were excluded.

### 2.3. Variables and Outcomes

The primary exposure was coded neurological comorbidity or complication burden, defined by the presence of ICD-10-CM codes corresponding to Elixhauser “other neurological disorders.” Primary outcomes included total hospital charges and length of stay (LOS), both log-transformed for regression modeling. Secondary outcomes included in-hospital mortality, hospital-acquired complications (HACs), septicemia, fluid and electrolyte disorders (FEDs) [22], COVID-19 coded during hospitalization (Field 21), and admission through the emergency department (ED) [20]. Outcomes were identified using ICD-10-CM codes and HCUP-defined variables [19,20].

### 2.4. Covariates

Covariates included patient age, sex, race/ethnicity (White, Black, Hispanic, Other), primary payer (Medicare, Medicaid, private insurance, self-pay/other), median household income quartile by ZIP code, rurality (urban vs. micropolitan), elective admission status, and comorbidity burden using the Elixhauser Comorbidity Index. Hospital-level variables included bed size, teaching status, and region. All covariates were selected a priori based on the clinical relevance and the prior literature [11,12].

### 2.5. Statistical Analysis

Descriptive statistics were used to compare patient and hospital characteristics between hospitalizations with and without NCs. Survey weights provided by HCUP were applied to generate nationally representative estimates [19]. Both unweighted discharge counts and survey-weighted national estimates were reported to distinguish the analytic sample size from nationally representative estimates. Continuous variables were compared using *t*-tests and categorical variables using chi-square tests. Multivariable generalized linear models with log-link functions were used to assess associations between NCs and continuous outcomes (charges and LOS), while logistic regression models were used for binary outcomes. All models accounted for survey design and clustering. For total hospital charges and length of stay, log-transformed outcomes were modeled, and exponentiated coefficients were reported as adjusted geometric mean ratios (aGMRs), where values greater than 1 indicate higher adjusted geometric mean outcomes among hospitalizations with neurological complications. Binary outcomes were modeled using survey-weighted logistic regression and reported as adjusted odds ratios (aORs) with 95% confidence intervals. We used generalized linear models with log-link for charges and LOS: E[log(Y)|X] = β_0_ + β_1_X_NC + Σγ_i Z_i (where Y is charges or LOS; X_NC is NC presence; Z_i are covariates). Logistic models were used for binary outcomes. Models incorporated NIS complex survey design via the R survey package (svyglm with svydesign accounting for strata, clusters, and weights; Taylor series linearization for variance). Multicollinearity was assessed (all variance inflation factors (VIFs) < 5). Model fit was evaluated and showed a satisfactory fit.

Statistical significance was defined as a two-sided *p*-value < 0.05. Analyses were conducted using R version 3.6.3.

## 3. Results

### 3.1. Cohort Characteristics

The analytic cohort included 11,523 unweighted hospitalizations, corresponding to 57,615 survey-weighted hospitalizations with a primary diagnosis of head and neck cancer (HNC). Of these, 1328 unweighted hospitalizations, corresponding to 6320 weighted hospitalizations (11.0%), involved neurological complications (NCs) (Table 1). Patients with NCs were older than those without NCs, with a mean age of 66.7 ± 11.9 versus 64.9 ± 12.1 years, respectively (*p* < 0.001), and less often female with 25.4% versus 28.2%, respectively (*p* = 0.042). Elective admissions were less frequent in the NC group (15.8% vs. 37.4%; *p* < 0.001). Patients with NCs had higher comorbidity burden, with mean Elixhauser scores of 23.9 ± 9.7 compared with 13.9 ± 9.9 among those without NCs (*p* < 0.001). Racial distribution differed significantly between groups (*p* < 0.001), with a higher proportion of Black patients in the NC cohort (14.6% vs. 9.9%). Insurance patterns also varied (*p* < 0.001): Medicare coverage was more common in NC hospitalizations (61.7% vs. 51.8%), and private insurance was less common (15.9% vs. 27.8%). Weekend admissions were more frequent in the NC group (19.9% vs. 14.9%; *p* < 0.001).

### 3.2. Unadjusted Outcomes

In unadjusted analyses, hospitalizations with neurological complications (NCs) had longer lengths of stay (10.1 vs. 6.5 days; *p* < 0.001) and higher total hospital charges ($140,702 vs. $101,697; *p* < 0.001) than those without NCs (Table 1). In-hospital mortality was higher in the NC group (15.2% vs. 3.9%; *p* < 0.001). Hospital-acquired complications occurred more frequently in NC hospitalizations (30.0% vs. 11.6%; *p* < 0.001). Septicemia was more common among patients with NCs (27.7% vs. 10.6%; *p* < 0.001). Fluid and electrolyte disorders occurred in 61.6% of NC admissions compared with 40.2% in non-NC admissions (*p* < 0.001). Patients with NCs were more-often admitted through the emergency department (73.4% vs. 51.1%; *p* < 0.001) and more-frequently admitted on weekends (19.9% vs. 14.9%; *p* < 0.001). COVID-19 coding during hospitalization was also more common among NC admissions (4.3% vs. 2.1%; *p* < 0.001).

### 3.3. Adjusted Analyses

After adjustment for available demographic, clinical, and hospital-level covariates, neurological complications (NCs) remained associated with increased healthcare utilization and adverse outcomes (Table 2). These associations should not be interpreted as causal because residual confounding by cancer severity, treatment intensity, and unmeasured clinical factors cannot be excluded.

**Figure 1:**
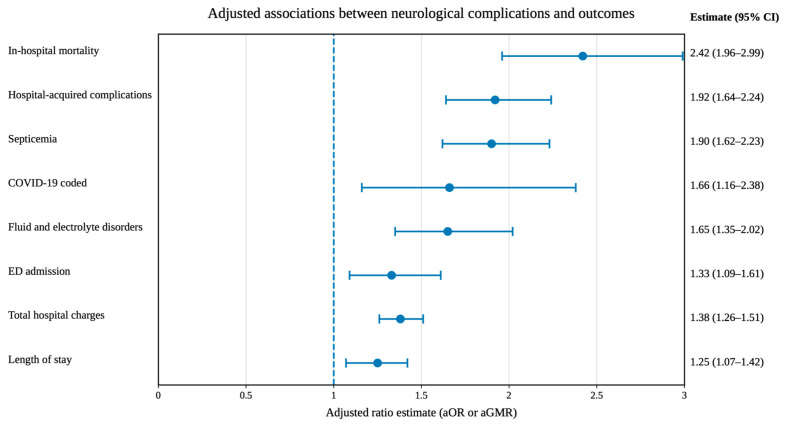
Adjusted associations between neurological disorders and clinical outcomes among hospitalized patients with head and neck cancer. Forest plot showing adjusted effect estimates for clinical outcomes associated with neurological disorders in hospitalized patients with head and neck cancer. Points represent adjusted effect estimates including adjusted odds ratios (aORs) for binary outcomes and adjusted geometric mean ratios (aGMRs) for total hospital charges and length of stay, and horizontal bars represent 95% confidence intervals. Neurological disorders were associated with increased in-hospital mortality, hospital-acquired complications, septicemia, fluid and electrolyte disorders, COVID-19 infection, emergency department admission, longer length of stay, and higher total hospital charges. Dashed vertical line at 1: the line of null effect (aOR or aGMR = 1.0).

NCs were associated with higher total hospital charges (aGMR, 1.38; 95% CI, 1.26–1.51) and longer length of stay (aGMR, 1.25; 95% CI, 1.07–1.42). NCs were also associated with higher odds of in-hospital mortality (aOR, 2.42; 95% CI, 1.96–2.99), hospital-acquired complications (aOR, 1.92; 95% CI, 1.64–2.24), septicemia (aOR, 1.90; 95% CI, 1.62–2.23), and fluid and electrolyte disorders (aOR, 1.65; 95% CI, 1.35–2.02). Additionally, NCs were associated with higher odds of COVID-19 coding during hospitalization (aOR, 1.66; 95% CI, 1.16–2.38) and admission through the emergency department (aOR, 1.33; 95% CI, 1.09–1.61) (Figure 1).

### 3.4. Disparities in Outcomes

Significant disparities were observed across race/ethnicity, insurance status, income level, and geographic residence (Table 3). Compared with White patients, Hispanic patients had higher adjusted hospital charges (aGMR, 1.35; 95% CI, 1.19–1.53), and patients categorized as Other racial groups had higher charges as well (aGMR, 1.26; 95% CI, 1.11–1.42). Black and Hispanic patients had longer adjusted length of stay than White patients (Black: aGMR, 1.25; 95% CI, 1.07–1.45; Hispanic: aGMR, 1.24; 95% CI, 1.03–1.49).

**Figure 2:**
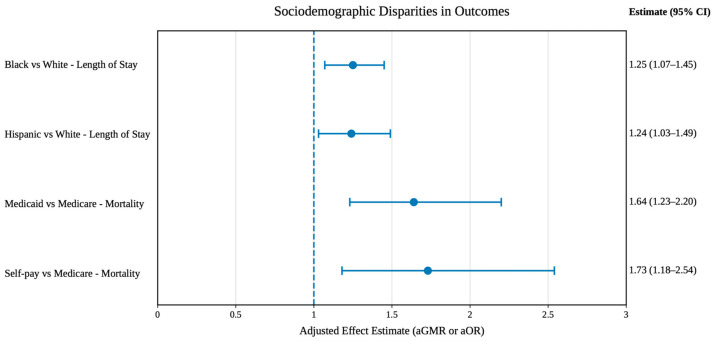
Disparities in clinical outcomes among hospitalized patients with head and neck cancer. Forest plot illustrating adjusted associations between race/ethnicity and payer status with selected hospital outcomes among patients hospitalized with head and neck cancer. Points represent adjusted effect estimates, including adjusted geometric mean ratios (aGMRs) for charges and length of stay and adjusted odds ratios (aORs) for binary outcomes, and horizontal bars represent 95% confidence intervals. Black and Hispanic patients experienced longer hospital length of stay compared with White patients. Patients insured by Medicaid or self-pay had higher in-hospital mortality compared with those insured by Medicare. Dashed vertical line at 1: the line of null effect (aOR or aGMR = 1.0).

Among hospitalizations with neurological complications, Medicaid-insured patients had higher adjusted odds of in-hospital mortality than Medicare beneficiaries (aOR, 1.64; 95% CI, 1.23–2.20), and self-pay/other insurance patients also had higher mortality (aOR, 1.73; 95% CI, 1.18–2.54) (Figure 2). Patients residing in micropolitan areas had lower adjusted odds of hospital-acquired complications than those in urban areas (aOR, 0.77; 95% CI, 0.63–0.93). Compared with hospitalizations from high-income ZIP code areas, lower-income ZIP codes had lower odds of documented COVID-19 during the hospitalization (aOR, 0.52; 95% CI, 0.34–0.77).

## 4. Discussion

In this nationally representative analysis of hospitalized patients with head and neck cancer (HNC), neurological complications (NCs) were independently associated with substantially greater resource utilization and worse clinical outcomes, including nearly 2.5-fold higher odds of in-hospital mortality. These findings should be interpreted as associations rather than evidence of direct causality, as the NIS does not capture cancer stage, tumor burden, performance status, treatment modality, radiation exposure, surgical complexity, or brain metastasis status.

These findings align with the broader oncology literature linking delirium and cancer-related cognitive impairment (CRCI) to prolonged hospitalization, postoperative complications, and excess mortality, and they underscore the vulnerability of HNC patients who frequently undergo complex surgery and multimodality treatment [5,6,8,9,23,24]. The elevated rate of ED admissions among patients with NCs aligns with prior evidence highlighting the central role of the emergency department in unplanned cancer care [10]. The observed differences in healthcare utilization, including longer length of stay and markedly higher total hospital charges among patients with NCs, may reflect the increased need for multidisciplinary evaluations, prolonged stabilization, and intensive monitoring that follows the onset of neurocognitive dysfunction. However, NCs may also function as a marker of underlying illness severity. Patients with advanced HNC, metastatic disease, brain involvement, more-intensive multimodal therapy, or complex surgical courses may be more likely to develop or have neurological conditions documented during hospitalization. Therefore, the observed associations may reflect both the clinical impact of neurological dysfunction and residual confounding by disease severity and treatment burden. This limitation is particularly relevant when interpreting the mortality association.

Multiple intersecting mechanisms likely underlie these associations. Acute inpatient stressors such as infection, metabolic disturbances, hypoxemia, and exposure to sedatives or opioids may act on baseline vulnerability related to pre-existing comorbidities and neurologic conditions, including both identified and previously unrecognized impairment. Neuroinflammation and treatment-related neurotoxicity can impair attention and executive function, delaying recognition of clinical deterioration and adherence to care plans [25,26,27,28,29,30]. Systemic inflammatory responses and treatment-related mucosal injury increase susceptibility to infection, consistent with our observation of higher septicemia among patients with NCs [7,31]. Systemic inflammation underlying fatigue syndromes may further heighten susceptibility to NCs in hospitalized patients [32]. The significantly higher rates of fluid–electrolyte disorders in patients with NCs may relate in part to treatment-related complications from radiotherapy with or without systemic therapy, such as mucositis and dysphagia [27,29]. Decreased oral intake, sedative exposure, dehydration, and catabolic stress may further contribute to these disturbances and precipitate or exacerbate neurological dysfunction [22]. The elevated odds of septicemia among patients with NCs align with historical and contemporary reports of bidirectional interactions between neurologic dysfunction and infection risk in hospitalized populations [7,31,33].

Clinical implications are immediate and actionable. Multicomponent delirium-prevention bundles such as frequent orientation, sleep optimization, early mobilization, careful titration of sedatives, and pain management may help reduce downstream complications in this population. Routine electrolyte surveillance and protocolized correction could address the markedly elevated rates of disturbance observed in NC patients. Similarly, early sepsis screening may mitigate the elevated risk of infectious complications identified in our analysis. For patients receiving neurotoxic therapies, anticipatory counseling, pre-treatment neurocognitive assessment, and consideration of neuroprotective strategies used in other oncology settings (e.g., memantine during brain radiotherapy) may warrant evaluation in future HNC-specific pathways [30,34,35,36].

Disparities in outcomes should be interpreted within both structural/institutional and geographic/case-mix contexts. Structural barriers may partly explain the higher charges observed among Hispanic and Other racial groups, longer LOS among Black and Hispanic patients, and higher mortality among Medicaid and self-pay patients. These patterns are unlikely to reflect biological differences; rather, they may reflect unequal access to timely specialty care, outpatient follow-up, language-concordant care, discharge planning resources, post-acute placement, and care navigation [11,12,37,38]. Differences in baseline comorbidity burden, including higher population-level prevalence of hypertension, diabetes mellitus, and obesity in some groups, may further increase vulnerability to inpatient complications [39,40,41]. Insurance-related mortality differences may also reflect delayed presentation, reduced access to pre-hospital stabilization, and limited longitudinal oncology support, all of which may increase vulnerability during hospitalization [18,42]. Similar insurance-related and structural access barriers have been reported in other healthcare settings, supporting the broader relevance of insurance status as a determinant of patient experience, access, and outcomes beyond the U.S. inpatient context [43].

From a policy and clinical practice perspective, these findings support universal, standardized screening for neurological complications and delirium, rather than selective clinician-dependent assessment that may introduce implicit bias. Early involvement of social work, culturally and linguistically responsive care managers, and patient navigation programs may help reduce discharge delays, improve post-acute care coordination, and mitigate financial toxicity among underinsured and minority patients.

Geographic findings should be interpreted separately. Patients residing in micropolitan areas had lower odds of hospital-acquired complications, which may reflect differences in case mix, coding practices, or thresholds for transfer to tertiary centers rather than true differences in underlying risk [12,44]. Therefore, geographic differences should not be interpreted as evidence of better outcomes in nonurban settings without further validation.

COVID-19 findings should also be interpreted cautiously. Variation in documented COVID-19 rates across income strata may also reflect differential access to testing, health care utilization patterns, or administrative coding practices during the pandemic rather than true differences in infection burden. In addition, the NIS does not distinguish community-acquired from hospital-acquired COVID-19 nor can it determine whether neurological manifestations preceded or followed infection. COVID-19 itself may contribute to delirium, encephalopathy, stroke, or other neurological manifestations, while patients with NCs may also be more vulnerable to infection due to frailty, prolonged hospitalization, and higher comorbidity burden [13,14,15,16].

The higher transfer-out status proportion among patients with NCs also suggests greater illness severity and care complexity. Transfers may reflect the need for tertiary-level services, specialized neurologic or oncologic management, complex airway care, rehabilitation, or post-acute placement. Because transfer status can influence both LOS and mortality, it may partially mediate or confound the observed associations between NCs and adverse outcomes. However, the NIS does not provide sufficient longitudinal information after transfer to determine downstream outcomes, timing of transfer, or whether transfer destination modified mortality risk. Therefore, transfer-related differences should be interpreted as an important marker of care complexity and residual confounding.

As survival among patients with head and neck cancer continues to improve, attention must increasingly shift toward long-term morbidity and survivorship outcomes. Neurological complications occurring during hospitalization may represent significant care needs and serve as early markers of vulnerability with implications that extend beyond the acute care setting, particularly in an aging population with substantial comorbidity burden. Prior studies have demonstrated that cancer-related neurocognitive impairment is associated with persistent functional decline, reduced quality of life, increased healthcare utilization, and challenges in treatment adherence during survivorship. In this context, neurological complications among hospitalized patients with HNC may influence recovery trajectories, functional independence, and the ability to engage in ongoing cancer care and rehabilitation. These findings underscore the importance of early recognition and management of neurocognitive syndromes during hospitalization, as well as the need for coordinated, multidisciplinary survivorship care models that integrate neurologic, oncologic, and supportive services to mitigate long-term adverse outcomes.

Strengths of this study include the use of the 2021 National Inpatient Sample, a large, nationally representative dataset, and the application of survey-weighted multivariable modeling to adjust for clinical, demographic, and hospital-level factors [19].

### 4.1. Future Directions

Future research should evaluate pragmatic inpatient care bundles tailored to HNC patients with NCs, incorporating early neurology or palliative care consultation, structured delirium prevention, protocolized electrolyte management, and sepsis early-warning tools. Health-system interventions addressing insurance-related barriers, travel burden, and rural access will also be essential to reduce inequities and improve outcomes at scale [11,12,37]. Additionally, future studies using longitudinal databases such as TriNetX may further elucidate the long-term neurological and survivorship-related complications associated with head and neck cancers and their treatments.

### 4.2. Limitations

This study has limitations. First, the use of administrative claims data from the National Inpatient Sample leads to the possibility of misclassification or under-coding of neurological complications, delirium severity, and comorbid conditions. The cross-sectional design precludes causal inference and does not allow evaluation of temporal relationships between neurological complications and downstream outcomes. Second, the NIS lacks key clinical variables, including tumor stage, tumor burden, metastatic disease, brain metastasis status, treatment modality, radiation exposure, chemotherapy, surgical complexity, performance status, and laboratory measures, and goals-of-care status. These unmeasured factors could influence both the likelihood of neurological complication coding and inpatient outcomes. Therefore, residual confounding by disease severity and treatment intensity may partially explain the observed associations, particularly for mortality, LOS, and hospital charges. Third, we were unable to distinguish acute neurological changes from pre-existing cognitive impairment, and differentiation between community-acquired and hospital-acquired COVID-19 was not feasible [21]. Additionally, rare but severe neurologic complications related to airway management or emergent intubation may not be consistently captured because of the limitations inherent to coding [45]. Furthermore, transfer-out status was more common among patients with NCs, but the NIS does not capture detailed post-transfer outcomes, timing of transfer, or granular discharge–destination pathways sufficient to determine whether transfer patterns mediated differences in LOS or mortality. Finally, documentation practices, staffing patterns, and diagnostic thresholds vary across hospitals and may contribute to unmeasured heterogeneity. These constraints, common to large database studies, underscore the need for prospective validation with standardized delirium screening, linkage with cancer registries, and inclusion of patient-reported outcomes [24].

## 5. Conclusions

In conclusion, neurological complications in hospitalized head and neck cancer patients are associated with increased healthcare utilization and adverse clinical outcomes, including elevated mortality, septicemia, and fluid–electrolyte disturbances. These findings highlight the importance of implementing proactive inpatient approaches, including delirium prevention bundles, infection control measures, and electrolyte monitoring; however, these findings should be interpreted within the limitations of administrative data and should not be considered evidence of direct causality. Observed differences across racial groups, insurance categories, and geographic regions suggest the presence of systemic inequalities that warrant targeted, equity-oriented interventions and health policy attention. Future investigations should focus on the prospective validation of standardized neurocognitive screening strategies and the incorporation of multidisciplinary care models of targeted interventions aimed at improving patient outcomes while mitigating the economic burden in this vulnerable population.

## Figures and Tables

**Table 1:** Baseline characteristics of hospitalized patients with head and neck cancer with and without neurological complications.

	HNC Without NC	HNC with NC	*p*-Value
Unweighted *n*	10,195	1328	
Weighted *n*	51,295	6320	
AGE (mean (SD *))	64.87 (12.10)	66.72 (11.92)	<0.001
Sex (%)			0.04
Female	14,450.0 (28.2)	1605.0 (25.4)	
RACE (%)			0.67
White	38,135.0 (76.2)	4515.0 (73.1)	
Black	4945.0 (9.9)	900.0 (14.6)	
Hispanic	3420.0 (6.8)	430.0 (7.0)	
Asian & Others	3530.0 (7.1)	330.0 (5.3)	
Median household income (based on current year)			0.03
0–25th percentile	13,675.0 (27.2)	1915.0 (30.9)	
26th–50th percentile	12,660.0 (25.1)	1540.0 (24.9)	
51st–75th percentile	12,330.0 (24.5)	1445.0 (23.3)	
76th–100th percentile	11,690.0 (23.2)	1290.0 (20.8)	
Expected primary payer (%)			<0.001
Medicare	26,510.0 (51.8)	3885.0 (61.7)	
Medicaid	7200.0 (14.1)	1035.0 (16.4)	
Private insurance	14,240.0 (27.8)	1000.0 (15.9)	
Self-pay, no charge and other	3265.0 (6.4)	380.0 (6.0)	
Patient location: NCHS * urban–rural code (%)			0.04
“Central” counties of metro areas of ≥1 million population	14,160.0 (27.8)	1880.0 (29.9)	
“Fringe” counties of metro areas of ≥1 million population	13,360.0 (26.2)	1650.0 (26.3)	
Counties in metro areas of 250,000–999,999 population	10,415.0 (20.4)	1390.0 (22.1)	
Counties in metro areas of 50,000–249,999 population	8200.0 (9.4)	60.0 (9.8)	
Micropolitan counties and neither metropolitan or micropolitan counties	15,380.0 (17.6)	90.0 (14.8)	
Admission type (%)			<0.001
Elective	19,140.0 (37.4)	995.0 (15.8)	
Indicator of a transfer out of the hospital			<0.001
Transferred out	7635.0 (14.9)	1950.0 (30.9)	
Weighted Elixhauser score mean (SD)	13.89 (9.90)	23.88 (9.70)	<0.001
Length of stay (geometric mean)	6.51	10.12	<0.001
Total charge (geometric mean)	$101,696.70	$140,701.86	<0.001
Mortality (%)	2000.0 (3.9)	960.0 (15.2)	<0.001

* Abbreviations: SD, standard deviation; NCHS, National Center for Health Statistics. Note: Unweighted *n* values represent the actual NIS discharge records included in the analytic sample. Weighted *n* values, frequencies, percentages, means, and standard deviations represent national estimates derived using 2021 National Inpatient Sample discharge weights.

**Table 2:** Adjusted associations between neurological complications and health outcomes.

Outcome	Measure	Adjusted Estimate (95% CI)	Interpretation
Total hospital charges	aGMR	1.38 (1.26–1.51)	38% increase
Length of stay (LOS)	aGMR	1.25 (1.07–1.42)	25% longer
In-hospital mortality	aOR	2.42 (1.96–2.99)	More than doubled
Hospital-acquired complications (HACs)	aOR	1.92 (1.64–2.24)	92% higher
Septicemia	aOR	1.90 (1.62–2.23)	90% higher
Fluid & electrolyte disorders	aOR	1.65 (1.35–2.02)	65% higher
COVID-19 coded	aOR	1.66 (1.16–2.38)	66% higher
ED admission	aOR	1.33 (1.09–1.61)	33% higher

All estimates are adjusted for age, sex, race/ethnicity, insurance, income, rurality, admission type, Elixhauser comorbidity index, and hospital-level factors (size, region, teaching status). aGMR, adjusted geometric mean ratio; aOR, adjusted odds ratio; CI, confidence interval; ED, emergency department. aGMRs represent exponentiated coefficients from survey-weighted regression models of log-transformed outcomes. Percent change was calculated as (aGMR − 1) × 100.

**Table 3:** Sociodemographic disparities in adjusted health outcomes among HNC patients.

Subgroup	Outcome	Adjusted Estimate (95% CI)	Interpretation
Hispanic vs. White	Charges	1.35 (1.19–1.53)	35% higher
Other vs. White	Charges	1.26 (1.11–1.42)	26% higher
Black vs. White	LOS	1.25 (1.07–1.45)	25% longer
Hispanic vs. White	LOS	1.24 (1.03–1.49)	24% longer
Medicaid vs. Medicare	Mortality (NC only)	1.64 (1.23–2.20)	64% higher
Self-pay vs. Medicare	Mortality (NC only)	1.73 (1.18–2.54)	73% higher
Micropolitan vs. Urban	HAC	0.77 (0.63–0.93)	23% lower
Low vs. High income	COVID-19	0.52 (0.34–0.77)	48% lower

For charges and length of stay, adjusted estimates are adjusted geometric mean ratios (aGMRs). For mortality, hospital-acquired complications, and COVID-19 outcomes, adjusted estimates are adjusted odds ratios (aORs).

## Data Availability

The analysis used 2021 National Inpatient Sample (NIS) data available from the HCUP Central Distributor (https://www.hcup-us.ahrq.gov/, accessed on 15 April 2026), subject to the data use agreement and fee. Analysis code and derived outputs are available from the corresponding author upon reasonable request.
